# Whole genome sequencing of M.tuberculosis in Kazakhstan: preliminary data

**DOI:** 10.5195/cajgh.2013.121

**Published:** 2014-03-27

**Authors:** Ulykbek Kairov, Ulan Kozhamkulov, Saule Rakhimova, Ayken Askapuli, Maxat Zhabagin, Venera Bismilda, Leyla Chingissova, Zhaxybay Zhumadilov, Ainur Akilzhanova

**Affiliations:** 1 Center for Life Sciences, Nazarbayev University, Astana, Kazakhstan; 2 National Center for Problems of Tuberculosis, Almaty, Kazakhstan

**Keywords:** tuberculosis, genome sequencing, Kazakhstan

## Abstract

**Background:**

Tuberculosis is a major public health problem which infects one third of the world’s population, resulting in more than two million deaths every year. The emergence of whole genome sequencing (WGS) technologies as a primary research tool has allowed for the detection of genetic diversity in Mycobacterium tuberculosis (MTB) with unprecedented resolution. WGS has been used to address a broad range of topics, including the dynamics of evolution, transmission, and treatment. To our knowledge, studies involving WGS of Kazakhstani strains of M. tuberculosis have not yet been performed.

**Aim:**

To perform whole genome sequencing of M. tuberculosis strains isolated in Kazakhstan and analyze sequence data (first experience and preliminary data).

**Results:**

In the present report, we announce the whole-genome sequences of the two clinical isolates of Mycobacterium tuberculosis, MTB-489 and MTB-476, isolated from the Almaty region. These strains were part of a repository that was created during our project “Creating prerequisites of personalized approach in the diagnosis and treatment of tuberculosis, based on whole genome-sequencing of M. tuberculosis”. Two strains were isolated from sputum samples of patients P1 and P2. Phenotypically, two isolates were drug-susceptible M. tuberculosis. Sequence data was compared with the publicly available data on M. tuberculosis laboratory strain H37Rv and others. The sequencing of the strains was performed on a Roche 454 GS FLX+ next-generation sequencing platform using a standard protocol for a shotgun genome library. The whole genome sequencing was performed for two *M.tuberculosis* isolates MTB-476 and MTB-489. 96 M bp with an average read length of 520 bp, approximately 21.8X coverage and 104.2 M bp with an average read length of 589 bp and approximately 23.7X coverage were generated for the MTB-476 and MTB-489, respectively. The genome of MTB-476 consists of 257 contigs, 4204 CDS, 46 tRNAs and 3 rRNAs. MTB-489 has 187 contigs, 4183 CDS, 45 tRNAs and 3rRNAs.

**Conclusion:**

The results of genome assembling have been submitted into NCBI GenBank and are available for public access under the accession numbers AZBA00000000 and AZAZ00000000. These genome assemblies can be useful for comparative genome analysis and for identification of novel SNPs and gene variants in genomes of M.tuberculosis.

